# Modeling of linear programming and extended TOPSIS in decision making problem under the framework of picture fuzzy sets

**DOI:** 10.1371/journal.pone.0220957

**Published:** 2019-08-13

**Authors:** M. Sarwar Sindhu, Tabasam Rashid, Agha Kashif

**Affiliations:** Department of Mathematics University of Management and Technology, Lahore - 54770, Pakistan; Shandong University of Science and Technology, CHINA

## Abstract

Picture fuzzy sets (PFSs) are comparatively a new extension of fuzzy sets which describe the human opinions that has more answers like acceptance, rejection, neutral and desist, which cannot be correctly presented in fuzzy sets (FSs) and intuitionistic fuzzy sets (IFSs). The PFSs are categorized by three objects, the degree of belonging, the degree of neutral belonging and the degree of non- belonging such that the total of these three degrees must not be more than one. So far, there is no such work presented in the literature which deals with unknown weights of criteria based on PFSs. In the present work, we have developed a linear programming (LP) model to find the exact weights from the given constraints of weights for the criteria and construct a modified distance based on similarity measure between picture fuzzy sets. Then we have utilized this similarity measure to achieve the best option in the multiple criteria decision making (MCDM) problem. Lastly, two practical examples for the selection of alternatives are presented to compare the obtained results with the existing similarity measures.

## Introduction

Zadeh presented the idea of fuzzy sets [1] in the middle of 1960s, which has opened the new horizon for the researchers. Fuzzy sets (FSs) generally exhibit uncertainty and ambiguity in real life problems. Most of the specialists have focused on the extensions of fuzzy sets (FSs) and its applications. The idea of intuitionistic fuzzy sets (IFSs) was presented by Atanassov [2] in 1986, the one of the important extension of FSs. In 2013, Cuong [3] introduced a novel concept of picture fuzzy sets (PFSs) that answered the human’s opinions which consist of more than two answers like, yes, no, refusal and neutral. Casting a vote is an excellent example of such situations because the voters can be divided into four groups such as vote for, vote neutral, vote against and vote refusal. Later on, Cuong and Kreinovich [4] presented some operations for PFSs. The PFSs are described by three components, the level of belonging, the level of non-belonging and the neutral level. The characteristic of these components is that the sum of the three levels must not be more than one.

The recent developments of PFSs included: Singh [5] found out the correlation coefficient of PFSs, Son [6] developed the distance measure and applied it to picture fuzzy clustering and Wei [7] presented the cross-entropy measure of PFSs and then implemented it for multiple attribute decision making problems. Son et al. [8] introduced a novel fuzzy inference structure on PFSs to improve the induction execution of the conventional fuzzy derivation framework. Thong and Son [9, 10] connected a novel picture fuzzy grouping methods for complex information. Wei [11] displayed the picture fuzzy accumulation operators strategy and used it to multi-attribute decision making (MADM) for positioning of enterprise resource planning (ERP). Garg [12] presented a few actions on PFSs and utilized it to multiple criteria decision making (MCDM) issues.

Furthermore, Jana et al. [13] presented some aggregation operators called Dombi operators for PFSs situations and implemented these actions to MADM process. Ashraf et al. [14] introduced a novel concept of cubic picture fuzzy sets, the extended form of PFSs. Wang and Li [15] extended the hesitant fuzzy sets to picture hesitant fuzzy sets and use it in MCDM. Wang et al. [16] formulated a hybrid fuzzy multiple criteria decision making framework with picture fuzzy information to rank the risk features of energy performance contracting (EPC) projects. Wang et al. [17] developed a novel comparison technique between two distance measures under the probabilistic linguistic term sets (PLTSs). Wang et al. [18] used the picture fuzzy numbers (PFNs) data in muirhead mean and weighted muirhead mean operators for multiple attribute decision making (MADM) problems. Moreover, Wei and Zhang [19] utilize power aggregation operators and Bonferroni mean to develop some single-valued neutrosophic Bonferroni power aggregation operators and single-valued neutrosophic geometric Bonferroni power aggregation operators to choose the best strategic suppliers. Recently, Zhang et al. [20] developed the score, accuracy functions and action rules for picture 2-tuple linguistic numbers (P2TLNs), implemented these for multiple criteria group decision making by using evaluation based on distance from average solution (EDAS). Wei et al. [21] extended the Maclaurin symmetric mean (MSM) operator and the dual MSM operator to q—rung orthopair fuzzy sets and deliberated their some properties in detail. Peng [22] constructed the picture fuzzy ordered weighted geometric (PFOWG) operator and picture fuzzy induced OWG (PFIOWG) operators to examine the multiple attribute decision making problems under picture fuzzy information.

In our daily life, we all are required to make distinct decisions intentionally and unintentionally which make us DMs. The data we assemble are to enable us to get our goals accurate and efficient. Not all the data are helpful for enhancing our comprehension and decisions. Decision making, for which we accumulate most of our data, has turned into a mathematical science nowadays [23]. DMs are inclined to use MCDM approaches in order to cope with multiple criteria problems more effectively. MCDM is a field of operational research where alternatives are assessed to select the most suitable alternative that satisfies an ideal objective from a set of multiple and often conflicting criteria [24, 25]. MCDM plays a dominant role in decision making and operational research. It is a collection of strategies and methodology by which different and conflicting criteria can be joined into a decision process.

Linear programming (LP) [26] is utilized to get ideal answers for task investigations. Utilizing LP enables scientists to locate the best, the most conservative answer to an issue inside the majority of its restrictions, or limitations. In order to increase the proficiency, LP procedures are preferably applied in the fields of agriculture, engineering, transportation, manufacturing and energy. LP permits characterizing factors, discovering imperatives and developing the objective function, or what should be optimized. Many researchers applied the LP technique in their proposed work for example, Wang and Chen [27] presented a new MCDM method based on linear programming methodology which provided new score and accuracy function of interval-valued intuitionistic fuzzy values (IVIFVs), Su et al. [28] presented an input-output LP model, to study energy-economic recovery resilience of an economy, Aliyev [29] presented interval LP where the ambiguous location is termed by interval numbers and Wang and Chen [30] presented LP methodology and the extended TOPSIS method for interval-valued intuitionistic fuzzy numbers for the selection of the best alternative, which deals with two interval values: a belonging and a non belonging. Recently, Sindhu et al. [31] applied the LP model to calculate the unknown weights of the criteria and utilize these weights on a certain MCDM problem.

TOPSIS technique introduced by Hawang and Yoon [32], that handles the MCDM issues with crisp information and the extended TOPSIS strategy [33] are generally utilized by the DMs with regards to different extensions of fuzzy sets. Kuo [34] figured out a flaw in TOPSIS and modified it for the different ranking index. Zhoua et al. [35] improved the TOPSIS with weighted hesitant vague information. Tian et al. [36] used the best-worst method to evaluate the weights of criteria and then utilized these weights in TOPSIS to resolve the MCGDM issues under intuitionistic fuzzy environment.

PFSs are applied in such environment in which the opinions of the decision makers (DMs) have more answers: acceptance, neutral, rejection and refusal which can not be accommodated precisely in FSs and IFSs. In order to find the weights of criteria, LP model is modest and user friendly, and responds rapidly as compared to other techniques. TOPSIS plays an important role to rank the alternatives in different fields of MCDM problems. Inspired by the merits and advantages of LP model, TOPSIS, as far as we know, LP model has not been considered under the framework of PFSs. It is noteworthy that the decision making under PFSs environment may acquire more attention and further research. Thus, we extend the LP model for PFSs to calculate the weights of criteria and then use these weights in TOPSIS to obtain the best alternative from the information provided by the DMs in the form of PFSs.

The remaining of the paper is organized as: In Section 2, we briefly describe the core concepts of fuzzy sets, intuitionistic fuzzy set, picture fuzzy sets and some operations about the PFSs. In Section 3, we developed a modified distance formula and a similarity measure based on the distance measure for PFSs. Section 4 contains a proposed MCDM method based on picture fuzzy TOPSIS (PF-TOPSIS). In Section 5, we utilize PF-TOPSIS on the practical examples to analyze the experimental results of the proposed technique. A comprehensive comparative analysis is discussed in Section 6. Conclusions and the future work direction are given in the Section 7.

## Some basic concepts

In the present section, a brief overview is given about the basic ideas associated to fuzzy sets (FSs), intuitionistic fuzzy set (IFSs), picture fuzzy sets (PFSs) and some operations like union, intersection and complement of PFSs.

**Definition 1**. [1] Let *X* = {*x*_1_, *x*_2_, …, *x*_*n*_} be a universe of discourse set, then a fuzzy set A defined on *X* can be written as a collection of ordered pairs as:

$$\begin{array}{c} A=\{\left(x,\mu_{A}\left(x\right)\right)|x\in X\}, \end{array}$$

where $\mu_{A}\left(x\right)):X\to \left[0,1\right]$ is a membership function so that *x* ∈ *X* to the set A.

**Definition 2**. [2] Let *X* be a fixed set, an intuitionistic fuzzy set A on *X* is defined as:

$$\begin{array}{c} A=\{\langle x,\alpha_{A}\left(x\right),\beta_{A}\left(x\right)\rangle |x\in X\}, \end{array}$$

where $\alpha_{A}\left(x\right)$, $\beta_{A}\left(x\right)\in \left[0,1\right]$ are called the membership and non membership degrees of *x* ∈ *X* to the set A, respectively with the condition: $0\leq \alpha_{A}\left(x\right)+\beta_{A}\left(x\right)\leq 1$, for all *x* ∈ *X*.

For all *x* ∈ *X*, *γ*_*A*_(*x*) is called the degree of hesitancy of x∈A where $\gamma_{A}\left(x\right)=1-\alpha_{A}\left(x\right)-\beta_{A}\left(x\right)$.

**Definition 3**. [3] Let *X* = {*x*_1_, *x*_2_, …, *x*_*n*_} be a fixed set, a picture fuzzy set P on *X* is defined as:

$$\begin{array}{c} P=\{\langle x,\alpha_{P}\left(x\right),\gamma_{P}\left(x\right),\beta_{P}\left(x\right)\rangle |x\in X\}, \end{array}$$

where $\alpha_{P}\left(x\right)$, $\beta_{P}\left(x\right)$, $\gamma_{P}\left(x\right)\in \left[0,1\right]$ are called the acceptance membership, neutral and rejection membership degrees of *x* ∈ *X* to the set P, respectively and $\alpha_{P}\left(x\right)$, $\gamma_{P}\left(x\right)$ and $\beta_{P}\left(x\right)$ fulfil the condition: $0\leq \alpha_{P}\left(x\right)+\gamma_{P}\left(x\right)+\beta_{P}\left(x\right)\leq 1$, for all *x* ∈ *X*. Also $\eta_{P}\left(x\right)=1-\alpha_{P}\left(x\right)-\gamma_{P}\left(x\right)-\beta_{P}\left(x\right)$, then *η*_*P*_(*x*) is said to be a degree of refusal membership of *x* ∈ *X* in P. For our convenience, the picture fuzzy sets over a fixed set *X* is written as PFSs(X).

**Definition 4**. [4] Let A and B be two PFSs on *X*, then the union, intersection and complement are described as follows:

(1) A⊆B iff $\alpha_{A}\left(x\right)\leq \alpha_{B}\left(x\right)$, $\gamma_{A}\left(x\right)\leq \gamma_{B}\left(x\right)$ and $\beta_{A}\left(x\right)\geq \beta_{B}\left(x\right)$ such that for all *x* ∈ *X*;
(2) A=B iff A⊆B and B⊆A;
(3) $A⊔B=\{x,\max\left(\alpha_{A}\left(x\right),\alpha_{B}\left(x\right)\right),\min\left(\gamma_{A}\left(x\right),\gamma_{B}\left(x\right)\right),\min\left(\beta_{A}\left(x\right),\beta_{A}\left(x\right)\right)|x\in X\}$;
(4) $A⊓B=\{x,\min\left(\alpha_{A}\left(x\right),\alpha_{B}\left(x\right)\right),\min\left(\gamma_{A}\left(x\right),\gamma_{B}\left(x\right)\right),\max\left(\beta_{A}\left(x\right),\beta_{A}\left(x\right)\right)|x\in X\}$;
(5) $A^{C}=\left\{\langle x,\beta_{A}\left(x\right),\gamma_{A}\left(x\right),\alpha_{A}\left(x\right)\rangle |x\in X\right\}$.

**Definition 5**. [26]. A LP model is formulated as follows:

$$\begin{array}{ll} \text{Maximize:} & S=c_{1}x_{1}+c_{2}x_{2}+c_{3}x_{3}+\ldots +c_{n}x_{n}\\ \text{Subject to:} & a_{11}x_{1}+a_{12}x_{2}+a_{13}x_{3}+\ldots +a_{1n}x_{n}\leq b_{1}\\ & a_{21}x_{1}+a_{22}x_{2}+a_{23}x_{3}+\ldots +a_{2n}x_{n}\leq b_{2}\\ & \vdots \\ & a_{m1}x_{1}+a_{m2}x_{2}+a_{m3}x_{3}+\ldots +a_{mn}x_{n}\leq b_{m}\\ & x_{1},x_{2},\ldots ,x_{n}\geq 0, \end{array}$$

where *m* and *n* denotes the number of constraints and the number of decision variables *x*_1_, *x*_2_, …, *x*_*n*_. A solution (*x*_1_, *x*_2_, …, *x*_*n*_) is called feasible if it satisfies all of the constraints. The purpose of the LP methodology is to find the optimal values of the decision variables *x*_1_, *x*_2_, …, *x*_*n*_ for maximizing the linear function *S*.

The distance is a quite essential idea in the instinctive fuzzy set theory. It can reveal the variance between two instinctive fuzzy sets.

## A modified distance measure between PFSs

In the present section, we construct a modified distance measure between two PFSs by including an extra term, the neutral belonging degree term of the PFSs in the Wang and Xin’s formula [37].

**Definition 6**. Let A and B be two PFSs defined on a fixed set *X* = {*x*_1_, *x*_2_, …, *x*_*n*_}, then the distance $D_{p}\left(A,B\right)$ is defined as:

$$\begin{array}{c} \begin{array}{c} D_{p}\left(A,B\right)=\frac{1}{3n}\sum_{i=1}^{n}(\begin{array}{c} {}[|\alpha_{A}\left(x_{i}\right)-\alpha_{B}\left(x_{i}\right)|+|\gamma_{A}\left(x_{i}\right)-\gamma_{B}\left(x_{i}\right)|+|\beta_{A}\left(x_{i}\right)-\beta_{B}\left(x_{i}\right)|]+\\ \max[|\alpha_{A}\left(x_{i}\right)-\alpha_{B}\left(x_{i}\right)|,|\gamma_{A}\left(x_{i}\right)-\gamma_{B}\left(x_{i}\right)|,|\beta_{A}\left(x_{i}\right)-\beta_{B}\left(x_{i}\right)|] \end{array}) \end{array} \end{array}$$ (1)

**Example 1**. Let A and B be two PFSs defined on a set *X* = {*x*_1_, *x*_2_, *x*_3_} given by $A=\{\left(x_{1},\left(0.8,0.1,0\right)\right),\left(x_{2},\left(0.4,0.2,0.3\right)\right),\left(x_{3},\left(0.5,0.3,0\right)\right)\}$ and $B=\{\left(x_{1},\left(0.3,0.3,0.2\right)\right),\left(x_{2},\left(0.7,0.1,0.1\right)\right),\left(x_{3},\left(0.4,0.3,0.2\right)\right)\}$, then by using the distance formula defined in Definition 6, we get, $D_{p}\left(A,B\right)=0.2556$.

**Theorem 1**. Suppose that, D is a mapping D:PFSs(X)×PFSs(X)→[0,1], then

$D_{p}\left(A,B\right)$ is a distance measure if the conditions below hold:

(1) $0\leq D_{p}\left(A,B\right)\leq 1$;
(2) $D_{p}\left(A,B\right)=0$ iff A=B;
(3) $D_{p}\left(A,B\right)=D_{p}\left(B,A\right)$;
(4) $D_{p}\left(A,C\right)\geq D_{p}\left(A,B\right)$ and $D_{p}\left(A,C\right)\geq D_{p}\left(B,C\right)$, for any A,B,C∈PFSs(X).

**Proof**. As, (1)–(3) are obvious, we thereby, prove the last condition (4). For any *A*, *B*, *C* ∈ *PFSs*(*X*), and *A* ⊆ *B* ⊆ *C*, then based on Definition 6, we see that

$$\begin{array}{cc} & |\alpha_{A}\left(x_{i}\right)-\alpha_{C}\left(x_{i}\right)\left|\geq \right|\alpha_{A}\left(x_{i}\right)-\alpha_{B}\left(x_{i}\right)| \end{array}$$ (2)

$$\begin{array}{cc} & |\alpha_{A}\left(x_{i}\right)-\beta_{C}\left(x_{i}\right)\left|\geq \right|\alpha_{A}\left(x_{i}\right)-\beta_{B}\left(x_{i}\right)| \end{array}$$ (3)

$$\begin{array}{cc} & |\alpha_{A}\left(x_{i}\right)-\gamma_{C}\left(x_{i}\right)\left|\geq \right|\alpha_{A}\left(x_{i}\right)-\gamma_{B}\left(x_{i}\right)| \end{array}$$ (4)

By adding Eqs (3)–(5), we get

$$\begin{array}{cc} & |\alpha_{A}\left(x_{i}\right)-\alpha_{C}\left(x_{i}\right)\left|+\right|\alpha_{A}\left(x_{i}\right)-\beta_{C}\left(x_{i}\right)\left|+\right|\alpha_{A}\left(x_{i}\right)-\gamma_{C}\left(x_{i}\right)|\\ & \geq \alpha_{A}\left(x_{i}\right)-\alpha_{B}\left(x_{i}\right)\left|+\right|\alpha_{A}\left(x_{i}\right)-\beta_{B}\left(x_{i}\right)\left|+\alpha_{A}\left(x_{i}\right)-\gamma_{B}\left(x_{i}\right)\right| \end{array}$$

⇒

$$\begin{array}{cc} & |\alpha_{A}\left(x_{i}\right)-\alpha_{C}\left(x_{i}\right)\left|+\right|\alpha_{A}\left(x_{i}\right)-\beta_{C}\left(x_{i}\right)\left|+\right|\alpha_{A}\left(x_{i}\right)-\gamma_{C}\left(x_{i}\right)\left|+\max\{\right|\alpha_{A}\left(x_{i}\right)-\alpha_{C}\left(x_{i}\right)|,\\ & |\alpha_{A}\left(x_{i}\right)-\beta_{C}\left(x_{i}\right)\left|,\right|\alpha_{A}\left(x_{i}\right)-\gamma_{C}\left(x_{i}\right)\left|\}\geq \right|\alpha_{A}\left(x_{i}\right)-\alpha_{B}\left(x_{i}\right)\left|+\right|\alpha_{A}\left(x_{i}\right)-\beta_{B}\left(x_{i}\right)|\\ & +|\alpha_{A}\left(x_{i}\right)-\gamma_{B}\left(x_{i}\right)\left|+\max\{\right|\alpha_{A}\left(x_{i}\right)-\alpha_{B}\left(x_{i}\right)\left|,\right|\alpha_{A}\left(x_{i}\right)-\beta_{B}\left(x_{i}\right)\left|,\right|\alpha_{A}\left(x_{i}\right)-\gamma_{B}\left(x_{i}\right)\left|\right\} \end{array}$$

⇒ $D_{P}\left(A,C\right)\geq D_{P}\left(A,B\right)$, on the same way, we can show that, $D_{P}\left(A,C\right)\geq D_{P}\left(B,C\right)$.

Commonly, the weights of the criteria have significant features in decision making, so we formulate the distance measure presented in Definition 6 into the weighted distance measure between two PFSs as:

**Definition 7**. Let A and B be two PFSs defined on a fixed set *X* = {*x*_1_, *x*_2_, …, *x*_*n*_} and *w*_*j*_ be the weights of the *m* criteria such that $\sum_{j=1}^{m}w_{j}=1$. Then the weighted distance measure $D_{p}^{w}\left(A,B\right)$ is defined as

$$\begin{array}{c} \begin{array}{c} D_{p}^{w}\left(A,B\right)=\frac{1}{3}\sum_{i=1}^{n}w_{j}(\begin{array}{c} {}[|\alpha_{A}\left(x_{i}\right)-\alpha_{B}\left(x_{i}\right)|+|\gamma_{A}\left(x_{i}\right)-\gamma_{B}\left(x_{i}\right)|+|\beta_{A}\left(x_{i}\right)-\beta_{B}\left(x_{i}\right)|]+\\ \max[|\alpha_{A}\left(x_{i}\right)-\alpha_{B}\left(x_{i}\right)|,|\gamma_{A}\left(x_{i}\right)-\gamma_{B}\left(x_{i}\right)|,|\beta_{A}\left(x_{i}\right)-\beta_{B}\left(x_{i}\right)|] \end{array}) \end{array} \end{array}$$ (5)

**Example 2**. Let A and B be two PFSs on a set *X* = {*x*_1_, *x*_2_, *x*_3_} described in Example 1 and the weights of *x*_1_, *x*_2_ and *x*_3_ are *w*_1_ = 0.25, *w*_2_ = 0.35 and *w*_3_ = 0.4, respectively. Hence the weighted distance between A and B by using Definition 7 is $D_{p}^{w}\left(A,B\right)=0.2883$.

**Theorem 2**. Let *X* = {*x*_1_, *x*_2_, …, *x*_*n*_} be a fixed set, then $D_{p}^{w}\left(A,B\right)$ is the level of weighted distance measure between two PFSs A and B satisfy the following conditions:

(1) $0\leq D_{p}^{w}\left(A,B\right)\leq 1$;
(2) $D_{p}^{w}\left(A,B\right)=0$ iff A=B;
(3) $D_{p}^{w}\left(A,B\right)=D_{p}\left(B,A\right)$;
(4) $D_{p}^{w}\left(A,C\right)\geq D_{p}^{w}\left(A,B\right)$ and $D_{p}^{w}\left(A,C\right)\geq D_{p}^{w}\left(B,C\right)$, for any A,B,C∈PFSs(X).

**Proof**. Follow the same procedure as Theorem 1.

**Definition 8**. Let A and B be two PFSs defined on a fixed set *X* = {*x*_1_, *x*_2_, …, *x*_*n*_}. Then a similarity measure $S_{p}\left(A,B\right)$ based on Definition 7 is defined as

$$\begin{array}{c} \begin{array}{c} S_{p}\left(A,B\right)=1-\frac{1}{3}\sum_{i=1}^{n}w_{j}(\begin{array}{c} {}[|\alpha_{A}\left(x_{i}\right)-\alpha_{B}\left(x_{i}\right)|+|\gamma_{A}\left(x_{i}\right)-\gamma_{B}\left(x_{i}\right)|+|\beta_{A}\left(x_{i}\right)-\beta_{B}\left(x_{i}\right)|]+\\ \max[|\alpha_{A}\left(x_{i}\right)-\alpha_{B}\left(x_{i}\right)|,|\gamma_{A}\left(x_{i}\right)-\gamma_{B}\left(x_{i}\right)|,|\beta_{A}\left(x_{i}\right)-\beta_{B}\left(x_{i}\right)|] \end{array}) \end{array} \end{array}$$ (6)

where *w*_*j*_(1 ≤ *j* ≤ *m*) denotes the weights of the *m* criteria such that $\sum_{j=1}^{m}w_{j}=1$.

**Definition 9**. A mapping S:PFSs(X)×PFSs(X)→[0,1]. $S_{p}\left(A,B\right)$ is said to be a similarity measure if, $S_{p}\left(A,B\right)$ satisfy the conditions below:

(1) $0\leq S_{p}\left(A,B\right)\leq 1$;
(2) $S_{p}\left(A,B\right)=1$ iff A=B;
(3) $S_{p}\left(A,B\right)=S_{p}\left(B,A\right)$;
(4) $S_{p}\left(A,C\right)\leq S_{p}\left(A,B\right)$ and $S_{p}\left(A,C\right)\leq S_{p}\left(B,C\right)$, for any A,B,C∈PFSs(X) and A⊆B⊆C.

## Picture fuzzy TOPSIS (PF-TOPSIS) for MCDM

In this section, we proposed an MCDM with picture fuzzy information based on TOPSIS by using LP metrology, LP model is adopted to evaluate the weights of criteria under various constraints. Let *A* = {*A*_1_, *A*_2_, …, *A*_*n*_} be a discrete set of alternatives, and *U* = {*U*_1_, *U*_2_, …, *U*_*m*_} be the collection of criteria with *w* = {*w*_1_, *w*_2_, …, *w*_*m*_}, where $\sum_{j=1}^{m}w_{j}=1$ as the weighing vector of the criteria *U*_*j*_ where *j* = 1, 2, 3, …, *m*. A picture fuzzy decision matrix denoted by $R={\left[\nabla_{ij}\right]}_{n\times m}={\left[\left(\alpha_{ij},\gamma_{ij},\beta_{ij}\right)\right]}_{n\times m}$ with *α*_*ij*_ as degree of acceptance, *γ*_*ij*_ degree of neutral and *β*_*ij*_ degree of rejection that the alternatives *A*_*i*_(*i* = 1, 2, …, *n*) fulfils respectively. In order to make the best decision, the procedure to find the MCDM is as follow:

Step 1 Developed a picture fuzzy decision matrix denoted by $R={\left[\nabla_{ij}\right]}_{n\times m}$ according to the given information presented by the DM.
Step 2 Figure out the picture fuzzy positive ideal solution (PFPIS), $\nabla_{p}^{+}$ and picture fuzzy negative ideal solution (PFNIS), $\nabla_{p}^{-}$ as:
  $$\begin{array}{c} \begin{array}{c} \nabla_{p}^{+}=\{(\alpha_{ij}^{+},\gamma_{ij}^{+},\beta_{ij}^{+})\}=(\begin{array}{c} \{(\max_{j}\left(\alpha_{ij}\right),\min_{j}\left(\gamma_{ij}\right),\min_{j}\left(\beta_{ij}\right))\}:U_{j}\in J_{1}\\ \{(\max_{j}\left(\alpha_{ij}\right),\max_{j}\left(\gamma_{ij}\right),\min_{j}\left(\beta_{ij}\right))\}:U_{j}\in J_{2} \end{array}) \end{array} \end{array}$$ (7)
  $$\begin{array}{c} \begin{array}{c} \nabla_{p}^{-}=\{(\alpha_{ij}^{-},\gamma_{ij}^{-},\beta_{ij}^{-})\}=(\begin{array}{c} \{(\min_{j}\left(\alpha_{ij}\right),\min_{j}\left(\gamma_{ij}\right),\max_{j}\left(\beta_{ij}\right))\}:U_{j}\in J_{1}\\ \{(\min_{j}\left(\alpha_{ij}\right),\max_{j}\left(\gamma_{ij}\right),\max_{j}\left(\beta_{ij}\right))\}:U_{j}\in J_{2} \end{array}) \end{array} \end{array}$$ (8)
  where *J*_1_ is a subset of benefit criteria and *J*_2_ is a subset of cost criteria, and *J*_1_ ∩ *J*_2_ = ∅.
Step 3 Calculate the degree of weighted similarity $S_{p_{i}}^{+}$ between PFPIS $\nabla_{p}^{+}$ and each alternative as well as the degree of weighted similarity $S_{p_{i}}^{-}$ between PFNIS $\nabla_{p}^{-}$ by using Eq (6), respectively:
  $$\begin{array}{c} \begin{array}{c} S_{wpi}^{+}\left(A_{i},\nabla_{p}^{+}\right)=1-\frac{1}{3}\sum_{j=1}^{m}w_{j}(\begin{array}{c} {}[|\alpha_{A}\left(x_{i}\right)-\alpha_{ij}^{+}|+|\gamma_{A}\left(x_{i}\right)-\gamma_{ij}^{+}|+|\beta_{A}\left(x_{i}\right)-\beta_{ij}^{+}|]+\\ \max[|\alpha_{A}\left(x_{i}\right)-\alpha_{ij}^{+}|,|\gamma_{A}\left(x_{i}\right)-\gamma_{ij}^{+}|,|\beta_{A}\left(x_{i}\right)-\beta_{ij}^{+}|] \end{array}) \end{array} \end{array}$$ (9)
  $$\begin{array}{c} \begin{array}{c} S_{wpi}^{-}\left(A_{i},\nabla_{p}^{-}\right)=1-\frac{1}{3}\sum_{j=1}^{m}w_{j}(\begin{array}{c} {}[|\alpha_{A}\left(x_{i}\right)-\alpha_{ij}^{-}|+|\gamma_{A}\left(x_{i}\right)-\gamma_{ij}^{-}|+|\beta_{A}\left(x_{i}\right)-\beta_{ij}^{-}|]+\\ \max[|\alpha_{A}\left(x_{i}\right)-\alpha_{ij}^{-}|,|\gamma_{A}\left(x_{i}\right)-\gamma_{ij}^{-}|,|\beta_{A}\left(x_{i}\right)-\beta_{ij}^{-}|] \end{array}) \end{array} \end{array}$$ (10)
  where, 1 ≤ *i* ≤ *n*.
Step 4 Based on Eqs (9) and (10), construct the model to find the objective function *Z* for the weights of criteria as:
  $$\begin{array}{cc} & Z=(S_{pi}^{+}\left(A_{i},\nabla_{p}^{+}\right)-S_{pi}^{-}\left(A_{i},\nabla_{p}^{-}\right)) \end{array}$$ (11)
Step 5 By solving the LP model presented in [26], we get the weights *w*_*j*_ of the criteria *U*_*j*_ where *j* = 1, 2, 3, …, *m*, so that the objective function *Z* obtained in Step 4 is maximized.
Step 6 Based on Eqs (9) and (10), calculate the degree of similarity $S_{p_{i}}^{+}$ and $S_{p_{i}}^{-}$ between each alternative and the elements obtained in PFPIS $\nabla_{p}^{+}$ and PFNIS $\nabla_{p}^{-}$, respectively.
Step 7 Evaluate the relative closeness ${CR}_{i}$ of alternative *A*_*i*_ with respect to the PFPIS $\nabla_{p}^{+}$ as:
  $$\begin{array}{cc} {CR}_{i} & =\frac{S_{p_{i}}^{+}}{S_{p_{i}}^{+}+S_{p_{i}}^{-}} \end{array}$$ (12)
  The larger the value of the relative closeness ${CR}_{i}$ of the alternatives with regard to the PFPIS $\nabla_{p}^{+}$ means that, we get the best alternative from different alternative *A*_*i*_, where 1 ≤ *i* ≤ *n*.

We consider two practical examples of MCDM problems from the literature [13] and [11] to show the visibility and benefits of the proposed method.

## Practical examples

In this section, two practical examples are established to implement the proposed MCDM approach under the environment of PFSs.

## Example 3

An organization wants to hire a technical firm to manage the technicalities of the organization. For this purpose, decision maker call five technicians $T=\{S_{{}_{1}},S_{2},S_{3},S_{4},S_{5}\}$ from different firms to set up an interview under the four criteria *C* = {*C*_1_, *C*_2_, *C*_3_, *C*_4_} ∈ *J*_1_, that is all these criteria are beneficial criteria such that:

*C*_1_ (advancement in technology), *C*_2_ (market potential), *C*_3_ (ability of vendors) and *C*_4_ (formation of employment and the innovations in technology and of science). The numerical data is adopted from [13]. To evade any conflict, the DM gave the weights to the criteria under some traits accordingly.

Subject to:

$$\begin{array}{c} \begin{array}{cc} & -0.3w_{1}+0.2w_{2}+0.5w_{3}+0.6w_{4}\leq 0.55;\\ & 0.2w_{1}-0.1w_{2}+0.2w_{3}-0.25+w_{4}\leq 0.26;\\ & 0.1w_{1}+0.2w_{2}-0.3w_{3}0.4+w_{4}\leq 0.3;\\ & w_{1}+w_{2}+w_{3}+w_{4}=1;\\ & 0.1\leq w_{1}\leq 0.2;\\ & 0\leq w_{2}\leq 0.1;\\ & 0.2\leq w_{3}\leq 0.3;\\ & 0.3\leq w_{4}\leq 0.4. \end{array} \end{array}$$ (13)

Step 1 A matrix R is constructed according to provided information provided by the DM under the PFSs environment in Table 1.
Step 2 Based on Eqs (7) and (8), evaluate the picture fuzzy positive ideal solution (PFPIS), $\nabla_{p}^{+}$ and picture fuzzy negative ideal solution (PFNIS), $\nabla_{p}^{-}$, respectively:
  $$\nabla_{p}^{+}=[(0.8800,0.0600,0.0300),(0.9000,0.0700,0.0300),(0.4000,0.3300,0.0500),(0.7200,0.1400,0.0300)];$$
  $$\nabla_{p}^{-}=[(0.5600,0.0600,0.1000),(0.0800,0.0700,0.2200),(0.0300,0.3300,0.1900),(0.0700,0.1400,0.0900)].$$
Step 3 Evaluate the level of similarity $S_{p_{i}}^{+}$ between PFPIS $\nabla_{p}^{+}$ and each alternative as well as the degree of similarity $S_{p_{i}}^{-}$ between PFNIS $\nabla_{p}^{-}$, respectively, by using Eqs (9) and (10).
  $$\begin{array}{c} \begin{array}{c} S_{pi}^{+}\left(A_{i},\nabla_{p}^{+}\right)=1-\frac{1}{3}\sum_{j=1}^{4}w_{j}(\begin{array}{c} {}[|\alpha_{A}\left(x_{i}\right)-\alpha_{ij}^{+}|+|\gamma_{A}\left(x_{i}\right)-\gamma_{ij}^{+}|+|\beta_{A}\left(x_{i}\right)-\beta_{ij}^{+}|]+\\ \max[|\alpha_{A}\left(x_{i}\right)-\alpha_{ij}^{+}|,|\gamma_{A}\left(x_{i}\right)-\gamma_{ij}^{+}|,|\beta_{A}\left(x_{i}\right)-\beta_{ij}^{+}|] \end{array}) \end{array} \end{array}$$ (14)
  $$\begin{array}{c} \begin{array}{c} S_{pi}^{-}\left(A_{i},\nabla_{p}^{-}\right)=1-\frac{1}{3}\sum_{j=1}^{4}w_{j}(\begin{array}{c} {}[|\alpha_{A}\left(x_{i}\right)-\alpha_{ij}^{-}|+|\gamma_{A}\left(x_{i}\right)-\gamma_{ij}^{-}|+|\beta_{A}\left(x_{i}\right)-\beta_{ij}^{-}|]+\\ \max[|\alpha_{A}\left(x_{i}\right)-\alpha_{ij}^{-}|,|\gamma_{A}\left(x_{i}\right)-\gamma_{ij}^{-}|,|\beta_{A}\left(x_{i}\right)-\beta_{ij}^{-}|] \end{array}) \end{array} \end{array}$$ (15)
  where, 1 ≤ *i* ≤ 5.
Step 4 Substituting the values of $S_{pi}^{+}$ and $S_{pi}^{-}$ obtained from Eqs (14) and (15) in Eq (11), construct a model to find the objective function *Z* as:
  $$\begin{array}{cc} Z & =(S_{pi}^{+}\left(A_{i},\nabla_{p}^{+}\right)-S_{pi}^{-}\left(A_{i},\nabla_{p}^{-}\right)) \end{array}$$
  where, 1 ≤ *i* ≤ 5, we get, *Z* = 0.0720*w*_1_ + 0.0413*w*_2_ − 0.0347*w*_3_ + 0.0247*w*_4_
Step 5 Based on the objective function *Z* obtained in Step 4 and the constraints given by the DM in system of Eq (13), solve the LP model by maximizing *Z* presented in [26] to get the exact weights of the criteria as:
  $$w_{1}=0.2000;w_{2}=0.1000;w_{3}=0.3000;w_{4}=0.4000.$$
Step 6 After substituting the weights of criteria obtained in Step 5, evaluate the level of similarity $S_{p_{i}}^{+}$ and $S_{p_{i}}^{-}$ between each alternative and the elements obtained in PFPIS $\nabla_{p}^{+}$ and PFNIS $\nabla_{p}^{-}$, respectively, which are:
  $$S_{p_{1}}^{+}=0.4007;S_{p_{2}}^{+}=0.3257;S_{p_{3}}^{+}=0.3637;S_{p_{4}}^{+}=0.2060;S_{p_{5}}^{+}=0.2333;$$
  $$S_{p_{1}}^{-}=0.8067;S_{p_{2}}^{-}=0.8017;S_{p_{3}}^{-}=0.7857;S_{p_{4}}^{-}=0.7573;S_{p_{5}}^{-}=0.7833.$$
Step 7 Based on Eq (12), evaluate the relative closeness ${CR}_{i}$ of alternative *A*_*i*_, where, 1 ≤ *i* ≤ 5, such that: ${CR}_{1}=0.3319$; ${CR}_{2}=0.2889$; ${CR}_{3}=0.3164$; ${CR}_{4}=0.2138$ and ${CR}_{5}=0.2295$, which gives the ranking order as: *S*_1_ ≻ *S*_3_ ≻ *S*_2_ ≻ *S*_5_ ≻ *S*_4_, shows that the best alternative is *S*_1_.

**Table 1:** PFSs matrix R given by the DM’s information.

	*C*_1_	*C*_2_	*C*_3_	*C*_4_
*S*_1_	(0.56, 0.34, 0.10)	(0.90, 0.07, 0.03)	(0.40, 0.33, 0.19)	(0.09, 0.79, 0.03)
*S*_2_	(0.70, 0.10, 0.09)	(0.10, 0.66, 0.20)	(0.06, 0.81, 0.12)	(0.72, 0.14, 0.09)
*S*_3_	(0.88, 0.09, 0.03)	(0.08, 0.10, 0.06)	(0.05, 0.83, 0.09)	(0.65, 0.25, 0.07)
*S*_4_	(0.80, 0.07, 0.04)	(0.70, 0.15, 0.11)	(0.03, 0.88, 0.05)	(0.07, 0.82, 0.05)
*S*_5_	(0.85, 0.06, 0.03)	(0.64, 0.07, 0.22)	(0.06, 0.88, 0.05)	(0.13, 0.77, 0.09)

## Example 4

Another practical example is established to implement the suggested MCDM approach under the PFSs environment. The information given in the article presented by Wei [11] is adopted. The same proposed MCDM technique is applied to evaluate the best alternative.

Follow the same Steps as given in Example 3, we get the values of $S_{p_{i}}^{+}$ and $S_{p_{i}}^{-}$, respectively as:

$$S_{p_{1}}^{+}=0.3350;S_{p_{2}}^{+}=0.3360;S_{p_{3}}^{+}=0.3753;S_{p_{4}}^{+}=0.2220;S_{p_{5}}^{+}=0.2757;$$

$$S_{p_{1}}^{-}=0.7987;S_{p_{2}}^{-}=0.8117;S_{p_{3}}^{-}=0.7890;S_{p_{4}}^{-}=0.7403;S_{p_{5}}^{-}=0.7787,$$

and the corresponding values of relative closeness are: ${CR}_{1}=0.2955$; ${CR}_{2}=0.2928$; ${CR}_{3}=0.3224$; ${CR}_{4}=0.2307$ and ${CR}_{5}=0.2615$, which gives the preference order as: *A*_3_ ≻ *A*_1_ ≻ *A*_2_ ≻ *A*_5_ ≻ *A*_4_ that is *A*_3_ is the desired alternative.

## Comparative analysis

**Fig 1:**
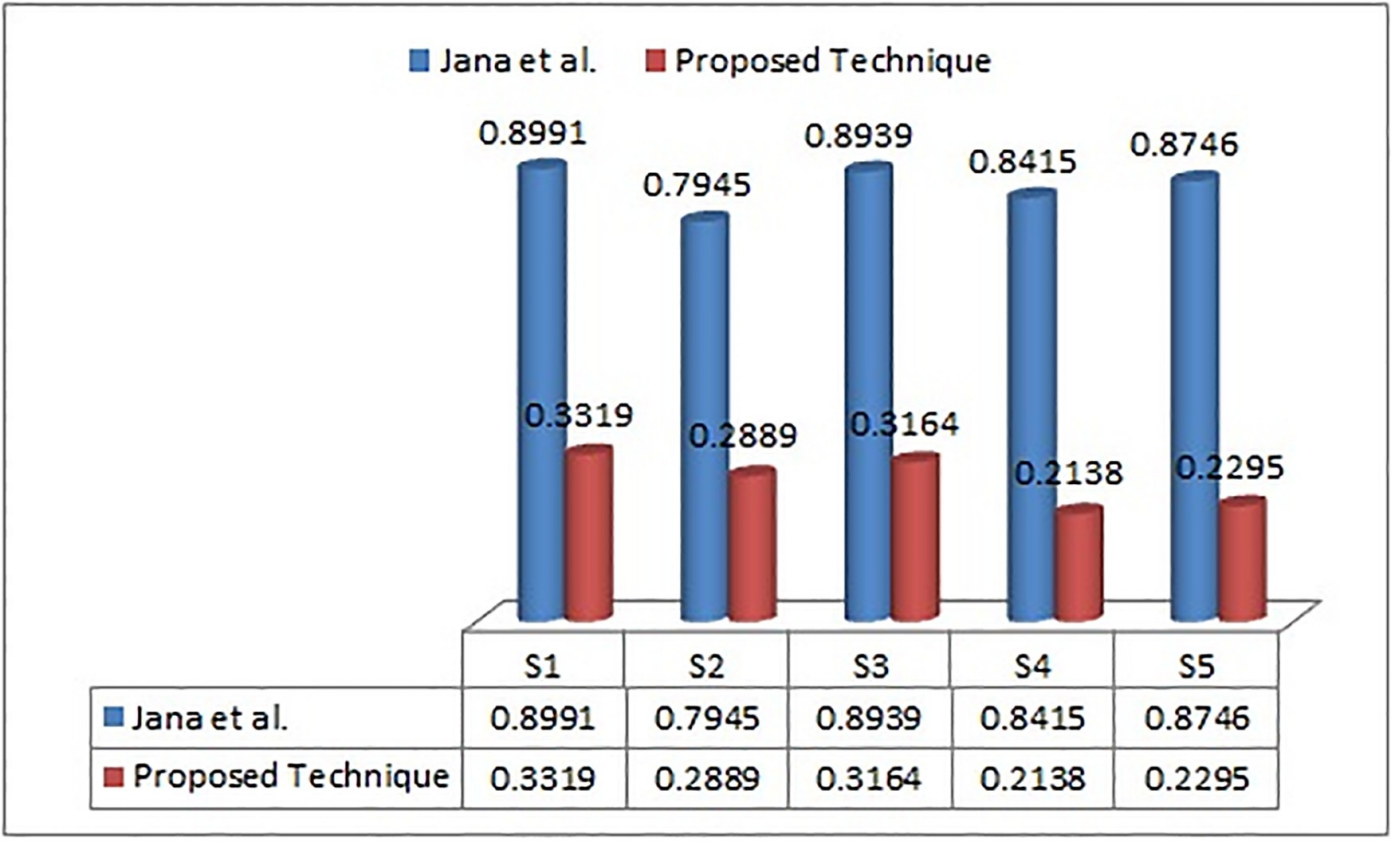
Graphical comparison of Jana et al. [13] and proposed technique.

**Fig 2:**
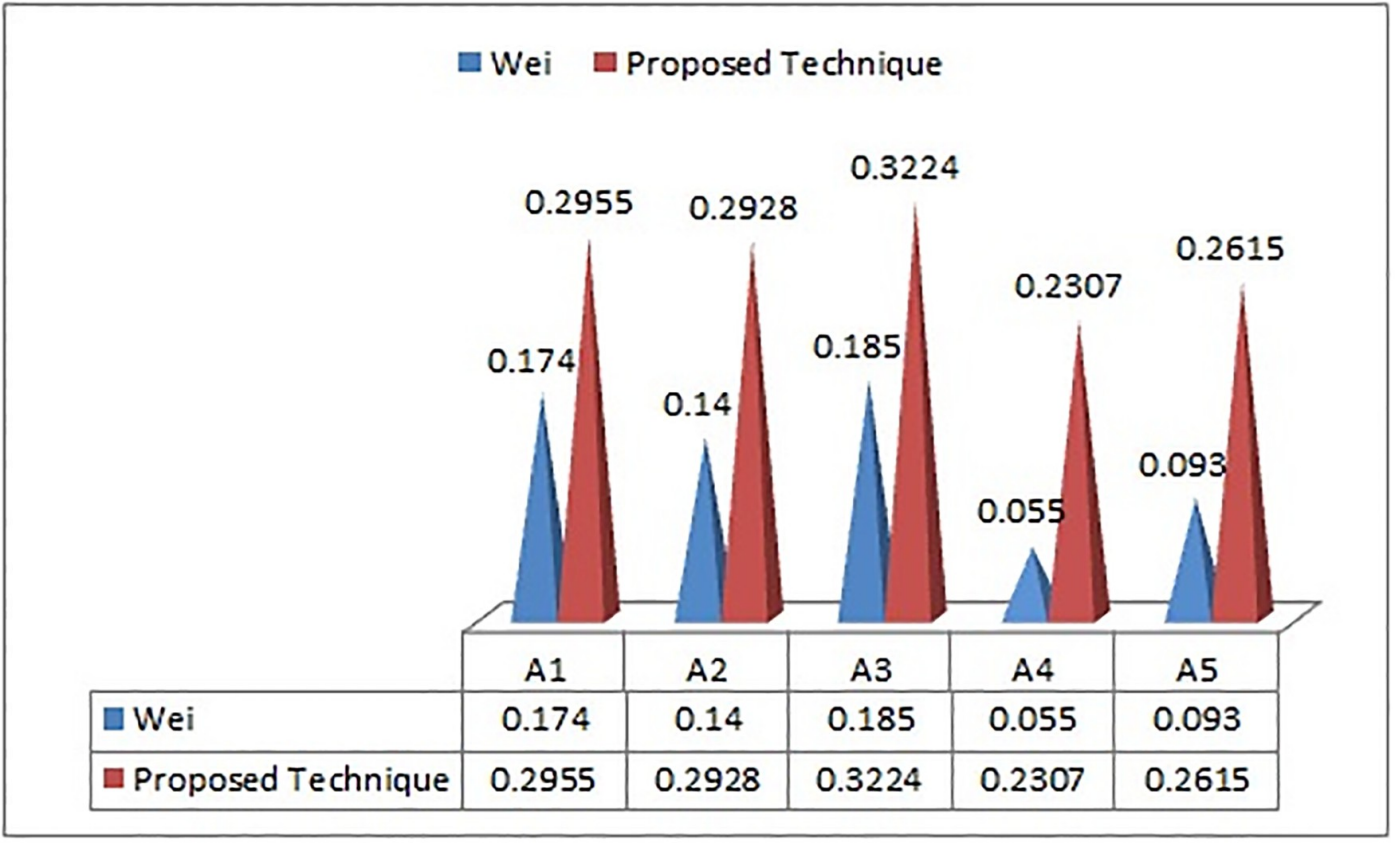
Graphical comparison of Wei and proposed technique.

In order to illustrate the strength and validity of the proposed method (PF-TOPSIS), we applied it on the information adopted from Jana et al. [13] and Wei [11]. The results obtained by using the proposed technique are then compared with existing methods. The technique proposed (PF-TOPSIS) in the present work deals with the picture fuzzy environment. The LP model is introduced to evaluate the unknown weights of criteria under the given constraints. The comparative results of the outcome achieved by the said technique with the Jana et al. [13] and Wei [11] are shown in Tables 2 and 3. In the practical Example 3, the preference order obtained by the proposed technique and picture fuzzy Dombi weighted average (PFDWA) presented by Jana et al. [13] are slightly different in arrangement but the desired best alternative is same, that is, *S*_1_ which shows the effectiveness of the proposed technique. However, in Example 4, the results obtained by the proposed technique and picture fuzzy weighted geometric (PFWG) operator provided by the Wei [11] are totally agreed to each other which also shows the usefulness of our proposed technique. Moreover, the techniques based on the aggregations have some limitations, like, its calculations are complex and hard. The complexity of evaluation can be increased rapidly if more elements are handled. But on the other hand, our proposed technique is based on the distance measure which is easy to calculate the intricate problems. The graphical representation of the preference order of the alternatives received by the proposed method, Wei [11] and Jana et al. [13] are shown in Figs 1 and 2.

**Table 2:** Comparison with Jana et al. [13] and proposed technique.

Techniques	${CR}_{1}$	${CR}_{2}$	${CR}_{3}$	${CR}_{4}$	${CR}_{5}$	Ranking arrangement
Jana et al. [13]	0.8991	0.7945	0.8939	0.8415	0.8746	*S*_1_ ≻ *S*_3_ ≻ *S*_5_ ≻ *S*_4_ ≻ *S*_2_
Proposed Technique	0.3319	0.2889	0.3164	0.2138	0.2295	*S*_1_ ≻ *S*_3_ ≻ *S*_2_ ≻ *S*_5_ ≻ *S*_4_

**Table 3:** Comparison with Wei [11] (PFWG) and proposed technique.

Techniques	${CR}_{1}$	${CR}_{2}$	${CR}_{3}$	${CR}_{4}$	${CR}_{5}$	Ranking arrangement
Wei [11] (PFWG)	0.174	0.140	0.185	0.055	0.093	*A*_3_ ≻ *A*_1_ ≻ *A*_2_ ≻ *A*_5_ ≻ *A*_4_
Proposed Technique	0.2955	0.2928	0.3224	0.2307	0.2615	*A*_3_ ≻ *A*_1_ ≻ *A*_2_ ≻ *A*_5_ ≻ *A*_4_

## Conclusions

Assigning the weights to the criteria is a difficult task for the DMs. Mostly, DMs feel hesitation or have less information to assign the weights to the criteria. LP technique is a useful tool that answers rapidly trough the MATLAB. In the present work, we focus to evaluate the weights of the criteria by using the linear programming model which is defined in Definition 5 that needs minimum appraisal information, yet leads to more reliable assessments as compared to other existing techniques. Furthermore, we utilized these weights in PF-TOPSIS to attain the best technical firm and enterprise resource planning (ERP) system. The comparative analysis exhibit the importance and efficiency of the suggested technique. The future research direction is to implement the suggested technique which can be extended in decision making problems under the framework of polygonal fuzzy sets and other vague situations.
